# Incidence of hypertension among persons living with HIV in China: a multicenter cohort study

**DOI:** 10.1186/s12889-020-08586-9

**Published:** 2020-06-01

**Authors:** Hongwei Fan, Fuping Guo, Evelyn Hsieh, Wei-Ti Chen, Wei Lv, Yang Han, Jing Xie, Yanling Li, Xiaojing Song, Taisheng Li

**Affiliations:** 1 Department of Infectious Diseases, Peking Union Medical College Hospital, Chinese Academy of Medical Sciences, Beijing, China; 2 Department of Internal Medicine, Yale School of Medicine, New Haven, CT USA; 3 School of Nursing, University of California, Los Angeles, CA USA; 4 Center for AIDS Research, Chinese Academy of Medical Sciences and Peking Union Medical College, Beijing, China

**Keywords:** HIV, Hypertension, Incidence

## Abstract

**Background:**

Life expectancy among persons living with HIV (PLWH) has improved with increasing access to antiretroviral therapy (ART), however incidence of chronic comorbidities has simultaneously increased. No data are available regarding the incidence of hypertension among Chinese PLWH.

**Methods:**

We analyzed data collected from patients enrolled in two prospective longitudinal multicenter studies of PLWH initiating ART in China. Incidence rate of hypertension per 100 person-years (PYs) among PLWH was calculated, and Cox proportional hazards models was used to evaluate the association between incident hypertension and traditional and HIV-associated risk factors.

**Results:**

Of 1078 patients included in this analysis, 984 ART-naïve patients were hypertension-free at baseline, and contributed 2337.7 PYs of follow up, with a median follow-up period of 1.8 years (range: 1.2–3.2) after initiation of ART. Incidence of hypertension was 7.6 [95% confidence interval (CI): 6.5–8.7] per 100 PYs. In the Cox regression analysis, incidence of hypertension was positively associated with body mass index [adjusted hazard ratio (aHR) 1.07 (1.01,1.13), *p* = 0.02] and recent viral load (aHR 1.28, 95% CI:1.08–1.51, *p* < 0.01), and negatively associated with recent CD4+/CD8+ ratio (aHR 0.14, 95% CI:0.06–0.31, *p* < 0.001), zidovudine exposure (aHR 0.15, 95% CI: 0.10–0.24, *p* < 0.001) and tenofovir disoproxil fumarate exposure (aHR 0.13, 95% CI: 0.08–0.21, p < 0.001).

**Conclusions:**

The incidence of hypertension was relatively high among Chinese PLWH initiating ART. Recent low CD4+/CD8+ ratio and detectable HIV viremia were associated with incident hypertension, whereas receipt of ART was associated with reduced risk. Hypertension may be mitigated, in part, by excellent HIV care, including viral suppression with ART.

**Trial registration:**

ClinicalTrials.gov Identifier: NCT00872417 registered on 31 March, 2009, and NCT01844297 registered on 1 May, 2013.

## Background

Due to the increased life expectancy of persons living with HIV (PLWH) after successful antiretroviral therapy (ART), management of aging-related non-communicable diseases (including cardiovascular disease, diabetes, chronic kidney disease, osteoporosis, and non-AIDS malignancies) has become a routine part of HIV care [1]. Hypertension is a key risk factor for cardiovascular diseases and is prevalent among PLWH [2]. A recent meta-analysis, including 49 studies with 63,554 participants from America, Europe, Africa and Asia from 1996 to 2014, reported an estimated hypertension prevalence of 12.7% for ART-naïve and 34.7% for ART-experienced participants [3]. Epidemiological studies from the United States and several European countries have demonstrated incidence of hypertension among outpatient primary care and HIV clinics for PLWH ranging from 2.6 to 7.2 per 100 person-years (PYs) [4–9]. Studies from East Africa have reported incidence rates of 11.2–12.0 per 100 PYs [10, 11].

Limited studies have focused on hypertension prevalence among PLWH in Asia [12–14], and these have all been cross-sectional in design. We previously reported the prevalence of hypertension was 8.4% among Chinese ART-naïve PLWH [12], which is lower than that reported in the USA and Europe [3]. However, the incidence of hypertension among PLWH in Asia, particularly after initiation of ART, remains unclear.

The risk factors contributing to hypertension among PLWH are multifactorial, controversial, and include traditional contributors (e.g. older age, male sex, obesity, family history, smoking, comorbidities) [5–8, 10, 11], and, in some studies, HIV-related factors including immunodeficiency [11], longer duration of HIV infection or advanced HIV clinical stage [4, 6], and ART [15]. However, other studies have reported no association between HIV-related factors and hypertension risk among ART-experienced PLWH [8, 10].

In order to provide important data regarding hypertension incidence among Chinese PLWH after initiation of ART, and to examine the association of traditional and HIV-associated risk factors with incident hypertension in this population, we designed the present analysis, leveraging data collected as part of two large prospective multicenter studies among Chinese PLWH.

## Methods

### Study Design & Population

We performed a secondary analysis of data collected as part of two large prospective multicenter studies of adult patients with HIV in China carried out by the same collaborative research network. These studies recruited patients from regions of high HIV prevalence across China, and were established to systematically evaluate the efficacy, toxicities and co-morbidities associated with the first-line government-sponsored free ART regimens available at the time each study was initiated.

In brief, the first study, [China AIDS Clinical Trial (CACT) 1810; clinicaltrials.gov ID: NCT00872417], initiated in 2008, was carried out in 8 cities across China (Beijing, Shanghai, Zhengzhou, Fuzhou, Guangzhou, Shenzhen, Xi’an and Yunnan), and recruited a total of 543 treatment-naïve adult patients with HIV from November 8, 2008 to August 6, 2010 [16]. This was an open-label trial comparing the efficacy and safety of three different ART treatment regimens over 96 weeks using government-sponsored first-line agents in 2008: Stavudine (d4T, 30 mg twice daily, Desano, Shanghai, China) or Zidovudine (AZT, 300 mg twice daily, Northeast General Pharmaceutical Factory, China) plus lamivudine (3TC, 300 mg once daily, GlaxoSmithKline, UK) and nevirapine (NVP, 200 mg once daily for the first 2 weeks and then 200 mg twice daily, Desano) or efavirenz (EFV, 600 mg once daily, MSD, Australia). Subsequent funding enabled an extension of this study beyond the initial 96-week follow-up period through the time of the present analysis.

The second study, (CACT1215 clinicaltrials.gov ID: NCT01844297), initiated in 2012, was carried out in 9 cities across China (Beijing, Shanghai, Guangzhou, Chengdu, Changsha, Nanning, Liuzhou, Zhengzhou, Shenyang) and recruited a total of 583 treatment-naïve adult patients with HIV from July 17, 2012 to July 3, 2014. This study was a cohort study examining the efficacy and safety at 96 weeks of the current first-line ART treatment regimen in China [tenofovir disoproxil fumarate (TDF, 300 mg once daily, Gilead Sciences, Inc., USA) plus 3TC and EFV]. Subsequent funding also allowed an extension of this study beyond the initial 96-week follow up period through the time of the present analysis.

Both studies collected sociodemographic data, clinical data related to HIV and HIV-related risk factors, and serologic biospecimens. Eligibility criteria at baseline included age 18–65 years, confirmed HIV-infection status via Western blot, CD4+ cell count < 350 cells/mm^3^ (for CACT1810) or < 500 cells/mm^3^ (for CACT1215), and lack of prior exposure to ART. After enrollment, participants initiated treatment with ART, and returned for follow-up evaluations at weeks 2, 4, 8, 12, 24, and once every 12 weeks thereafter for clinical and laboratory evaluations. Trained study staff at each study site performed detailed clinical evaluations and recorded data in an electronic study database.

Peking Union Medical College Hospital (PUMCH) in Beijing, China served as the primary study center for both studies, and training and monitoring of study sites was overseen by the same contract research organization. Both protocols were approved by the PUMCH institutional review board prior to initiation of study activities. All study participants provided written informed consent at the time of enrollment, and all procedures were performed in compliance with the ethical standards of *The Declaration of Helsinki*.

For the present analysis, we included patients enrolled in both parent studies in order to encompass ART treatment regimens utilized in China over the past decade. Patients who did not complete at least 2 visits (including the baseline visit) were excluded from our analysis (database cutoff date June 2015).

### Definitions

Hypertension was defined based upon the Joint National Committee on Prevention, Detection, Evaluation and Treatment of High Blood Pressure Recommendations [17], namely a systolic blood pressure ≥ 140 mmHg or diastolic blood pressure ≥ 90 mmHg at two different time points, or receiving a prescription for antihypertensive medication. Trained study personnel at each site performed standardized blood pressure measurements using a sphygmomanometer at each study encounter. For each participant, blood pressure was measured using a manual sphygmomanometer with appropriate cuff size based on arm circumference after more than 5 min of seated rest and without smoking, exercise or feeding. One measurement was taken positioned at the level of the heart, and recorded as the blood pressure value.

We classified body mass index (BMI) using the following categories: underweight < 18.5, normal 18.5–24.9, overweight 25–29.9, and obese ≥30.0 kg/m^2^ [18]; and dyslipidemia using the following categories: total cholesterol > 5.2 mmol/l, high-density lipoprotein cholesterol (HDL-c) < 1.0 mmol/l, low-density lipoprotein cholesterol > 4.1 mmol/l, or triglycerides > 1.7 mmol/l [19]. Participants were classified as diabetic if they had a prior diagnosis of diabetes, a fasting plasma glucose ≥7.0 mmol/l or were being treated with insulin or oral hypoglycemic agents [12]. Renal function was assessed by the estimated glomerular filtration rate (eGFR) using the formula from the Chronic Kidney Disease Epidemiology Collaboration [20], and categorized following the chronic kidney disease criteria [21].

Data from each study encounter regarding CD4+ and CD8+ T-cell levels and CD4+/CD8+ ratios were collected and we defined recent VL, recent CD4+ cell counts and recent CD4+/CD8+ ratio as the laboratory values obtained at the visit when hypertension was diagnosed, or, for those who did not develop hypertension, the last study encounter. HIV-1 viral load (VL) measurements, performed using the Cobas AmpliPrep/Cobas TaqMan real-time RT-PCR Assay (Roche, CA, USA), were also collected. Undetectable VL results were given the value of the detection limit (20 copies/ml). Complete and incomplete viral suppression were defined as HIV VL < 50 and < 400 copies/mL, respectively.

### Statistical analysis

We tabulated and reported descriptive data using means ± standard deviations and frequencies. The Student’s *t*-test for parametric continuous variables, Mann-Whitney U test for non-parametric continuous variables, and the Chi-squared test for categorical variables were used to compare the clinical characteristics between patients with and without hypertension. Incidence rates of hypertension per 100 PYs during the observation period were calculated. Stepwise Cox regression analysis was used to estimate hazard ratios (HR) with 95% confidence intervals (CI). Patients were censored at time of incident of hypertension, loss to follow-up, death, withdrawal from study during follow-up or end of study. The observation period began at the enrollment visit and ended at the date of hypertension diagnosis or the last clinical encounter for those who did not develop hypertension. Participants with hypertension at baseline were excluded from the longitudinal analyses.

Covariates analyzed included age, sex, BMI, Han ethnicity, current smoking, current alcohol use, eGFR, diabetes, dyslipidemia, hepatitis B virus (HBV) surface antigen (HBsAg), hepatitis C virus (HCV) antibody (HCV Ab), route of HIV transmission, years since HIV diagnosis, baseline CD4+ cell count, baseline VL, stavudine, zidovudine, and TDF exposure, recent CD4+ cell count, recent CD4+/CD8+ ratio, and recent VL. All statistical analyses were performed using SPSS 19.0 statistical software package (IBM Corporation, Armonk, New York, USA) and Prism version 6 (GraphPad Software, Inc., La Jolla, CA). For all tests, *p* < 0.05 was considered statistically significant.

## Results

### Population characteristics

**Fig. 1:**
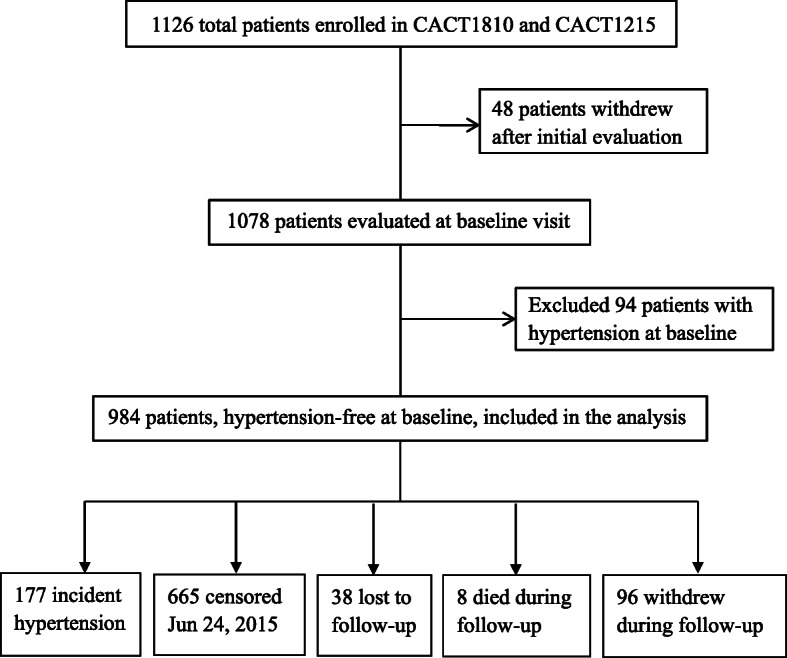
Patient flow chart. Abbreviation: CACT: China AIDS Clinical Trial

Out of 1126 total patients enrolled in the parent studies, we excluded forty-eight patients who withdrew after the initial evaluation, yielding 1078 patients eligible for analysis (Fig. 1). Of the included participants (mean age 35.7 ± 10.1 years, 75.0% men), Han Chinese comprised 84.9% of individuals, current smokers represented 23.9, 12.2 and 7.3% were co-infected with HBV and HCV respectively, and men who have sex with men (MSM) represented 39.4% of the population. The baseline CD4+ cell count was 234 ± 124 cells/mm^3^, and the baseline VL was 4.7 ± 0.7 log10 copies/ml (Table 1).

**Table 1:** Demographic and Clinical Characteristics, Stratified by Baseline Hypertension Status (*N* = 1078)

***Characteristic***	***Total*** ***(n = 1078)***	***No HTN*** ***(n = 984)***	***Baseline HTN*** ***(n = 94)***	***P value***
Male	808 (75.0)	740 (75.2)	68 (72.3)	0.541
Age (*years)*	35.7 ± 10.1	35.2 ± 9.9	41.0 ± 10.8	< 0.001
BMI (*kg/m*^*2*^*)*	21.6 ± 2.9	21.5 ± 2.9	22.8 ± 3.0	< 0.001
Overweight	113 (10.5)	93 (9.5)	20 (21.3)	< 0.001
Obese	11 (1.0)	10 (1.0)	1 (1.1)	NA
Han ethnicity	915 (84.9)	833 (84.7)	82 (87.2)	0.505
Current smoking	258 (23.9)	238 (24.2)	20 (21.3)	0.527
Current alcohol use	288 (26.7)	264 (26.8)	24 (25.5)	0.786
eGFR (*ml/min*)	113 ± 17	114 ± 17	108 ± 17	0.002
eGFR < 90 (*ml/min)*	98 (9.3)	83 (8.6)	15 (16.3)	0.015
Diabetes	51 (4.8)	49 (5.1)	2 (2.2)	0.21
TC > 5.2 *(mmol/l)*	111 (10.5)	102 (10.6)	9 (9.8)	0.81
TG > 1.7 (*mmol/l)*	313 (29.6)	283 (29.4)	30 (32.6)	0.51
HDL-c < 1.0 (*mmol/l)*	323 (30.9)	303 (31.8)	20 (21.7)	0.046
LDL-c > 4.1 *(mmol/l)*	18 (1.7)	14 (1.5)	4 (4.3)	NA
Dyslipidemia	541 (52.2)	497 (52.6)	44 (47.8)	0.38
HBs Ag+	127 (12.2)	110 (11.6)	17 (19.1)	0.04
HCV-Ab+	75 (7.3)	69 (7.3)	6 (6.6)	0.79
Route of HIV transmission				0.01
Heterosexual	497 (46.1)	451 (45.8)	46 (48.9)	
Homosexual	425 (39.4)	400 (40.7)	25 (26.6)	
Blood transfusion	53 (4.9)	45 (4.6)	8 (8.5)	
Others	103 (9.6)	88 (8.9)	15 (16.0)	
Years since HIV diagnosis	1.0 ± 1.7	1.0 ± 1.7	1.1 ± 1.6	0.72
CD4+ cell count (*cells/mm*^*3*^*)*	234 ± 124	233 ± 125	251 ± 114	0.18
< 200	413 (38.5)	384 (39.1)	29 (31.2)	0.19
200–350	461 (42.9)	413 (42.1)	48 (51.6)	
350–499	200 (18.6)	184 (18.8)	16 (17.2)	
Viral load (*log10 copies/ml)*	4.7 ± 0.7	4.7 ± 0.7	4.6 ± 0.7	0.15
< 5	571 (67.3)	517 (67.3)	54 (66.7)	0.91
≥ 5	278 (32.7)	251 (33.2)	27 (33.3)	

All characteristics measured at baseline

Values are shown as n (%) or mean ± standard deviation

Abbreviations: *HTN* hypertension; *BMI* body mass index; *NA*, not available; *eGFR*, estimated glomerular filtration rate; *TC* total cholesterol; *TG*, triglycerides; *HDL-c*, high-density lipoprotein cholesterol; *LDL-c*, low-density lipoprotein cholesterol; *HBsAg+*, hepatitis B surface antigen; *HCV-Ab*, hepatitis C antibody; *HIV*, human immunodeficiency virus

At baseline (Table 1), 94 participants had a diagnosis of hypertension [8.7, 95% CI 7.0–10.4%]. When compared with the 984 participants without hypertension, those with hypertension at baseline were older (41.0 vs. 35.2 years), had higher BMI levels (22.8 vs. 21.5 kg/m^2^), lower eGFR (108 vs. 114 ml/min), lower prevalence of HDL-c < 1.0 mmol/l (21.7% vs. 31.8%), and higher prevalence of HBsAg+ (19.1% vs. 11.6%) (All *p* < 0.05). In addition, patients with hypertension at baseline were much less likely to have acquired HIV via homosexual transmission as compared with other routes. Finally, no differences between the two groups were found with respect to years since HIV diagnosis, baseline CD4+ cell count and VL.

The 984 participants included in the longitudinal study (Table 1 and Table 2) had a median follow up duration of 1.8 years (interquartile range, 1.2, 3.2 years; range: 0.01–6.3 years), and the following baseline characteristics: mean age 35.2 ± 9.9 years, 75.2% male, 84.7% of Han ethnicity, 40.7% MSM and 24.2% current smokers. Baseline CD4+ cell count was 233 ± 125cells/mm^3^, and HIV VL was 4.7 ± 0.7 log10 copies/ml. The prevalence of participants co-infected with hepatitis B and hepatitis C were 11.6 and 7.3% respectively. At the time of hypertension diagnosis or the last encounter for those who did not develop hypertension, median CD4+ cell count and VL were 431 ± 203 cells/mm^3^ and 1.6 ± 0.7 log10 copies/ml respectively. Complete and incomplete viral suppression were achieved by 78.2 and 87.6% of participants, respectively.

**Table 2:** Demographic and Clinical Characteristics, Stratified by Incident Hypertension Status (N = 984)

***Characteristic***	***No HTN(n = 807)***	***HTN (n = 177)***	***P value***
Male	603 (74.7)	137 (77.4)	0.46
Age (*years)*	34.4 ± 9.4	38.8 ± 11.2	< 0.001
BMI (*kg/m*^*2*^*)*	21.3 ± 2.8	22.1 ± 3.1	< 0.01
Overweight	72 (8.9)	21 (11.9)	0.23
Obese	6 (0.7)	4 (2.3)	NA
Han ethnicity	676 (83.8)	157 (88.7)	0.10
Smoking	200 (24.8)	38 (21.5)	0.35
Current alcohol use	224 (27.8)	40 (22.6)	0.16
eGFR (*ml/min*)	114 ± 16	110 ± 19	< 0.01
eGFR < 90 (*ml/min*)	62 (7.7)	21 (11.9)	0.07
Diabetes	40 (5.0)	9 (5.1)	0.95
TC > 5.2 (*mmol/l*)	81 (10.0)	21 (11.9)	0.48
TG > 1.7 (*mmol/l*)	229 (28.4)	54 (30.5)	0.59
HDL-c < 1.0 (*mmol/l*)	240 (29.7)	63 (35.6)	0.17
LDL-c > 4.1 (*mmol/l*)	11 (1.4)	3 (1.7)	NA
Dyslipidemia	397 (49.2)	100 (56.5)	0.15
HBs Ag+	88 (10.9)	22 (12.4)	0.51
HCV Ab+	50 (6.2)	19 (10.7)	0.03
Route of HIV transmission			< 0.001
Heterosexual	339 (42.0)	61 (34.5)	
Homosexual	370 (45.8)	81 (45.8)	
Blood transfusion	23 (2.9)	22 (12.4)	
Others	75 (9.3)	13 (7.3)	
Years since HIV diagnosis	1.0 ± 1.7	1.1 ± 1.8	0.36
Baseline CD4+ cell count (*cells/mm*^*3*^*)*	238 ± 125	206 ± 124	< 0.01
Baseline HIV Viral load (*log10 copies/ml)*	4.7 ± 0.7	4.7 ± 0.7	0.52
Stavudine exposure	248 (30.7)	97 (54.8)	< 0.001
Years of stavudine exposure Zidovudine exposure	1.2 ± 1.1 316 (39.2)	0.6 ± 0.7 64 (36.2)	< 0.0010.46
Years of zidovudine exposure TDF exposure	3.3 ± 1.9 470 (58.2)	1.2 ± 1.1 54 (30.5)	< 0.001 < 0.001
Years of TDF exposure Recent CD4+ cell count (*cells/mm*^*3*^*)*	1.8 ± 0.7 453 ± 204	0.4 ± 0.5 330 ± 162	< 0.001 < 0.001
Recent CD4+/CD8+ ratio	0.7 ± 0.5	0.5 ± 0.3	< 0.001
Recent VL (*log10 copies/ml)*	1.5 ± 0.7	2.0 ± 0.7	< 0.001

Values are shown as n (%) or mean ± standard deviation

Abbreviations: *HTN* hypertension; *BMI* body mass index; *NA* not available; *eGFR*, estimated glomerular filtration rate; *TC* total cholesterol; *TG* triglycerides; *HDL-c* high-density lipoprotein cholesterol; *LDL-c*, low-density lipoprotein cholesterol; *HBsAg+*, hepatitis B surface antigen; *HCV-Ab* hepatitis C antibody; *HIV* human immunodeficiency virus; *VL* HIV-1 viral load; *TDF* tenofovir disoproxil fumarate

^*^ Unless stated otherwise, characteristics reported represent baseline characteristics

At the database cutoff date (Fig. 1), 142 (14.4%) participants were no longer being followed for the following reasons: 8 participants died (one car accident, one liver cirrhosis, one lactic acidosis, two with opportunistic infection, two with cerebral hemorrhage, and one with unknown cause of death), 96 participants had withdrawn from the study [16 participants with virologic failure, 9 participants experienced severe adverse events (one opportunistic infection, one toxoplasma encephalopathy, one hepatotoxicity, two with rash, four with bone marrow suppression), 71 participants voluntarily withdrew from the study], and 38 participants were lost to follow-up.

### Incidence of hypertension

The 984 study participants included in the longitudinal analysis contributed a total of 2337.7 PYs of follow-up. One hundred seventy-seven participants developed hypertension during the follow-up period, yielding an incidence of 7.6 (95% CI: 6.5–8.7) per 100 PYs. When stratified by cohort, a total of 476 patients from CACT1810 contributed 1549.95 PYs of follow-up (median follow-up time of 3.9 years) and 123 patients developed hypertension during this time. A total of 508 patients from CACT1215 contributed 787.72 PYs (median follow-up time of 1.8 years) and 54 patients developed hypertension. The incidence of hypertension was not significantly different between the participants in the two groups [7.9 (95% CI: 6.6–9.2) v. 6.9 (95% CI: 5.1–8.7) per 100 PYs, respectively (*p* = 0.35)].

### Risk factors for incident hypertension

**Fig. 2:**
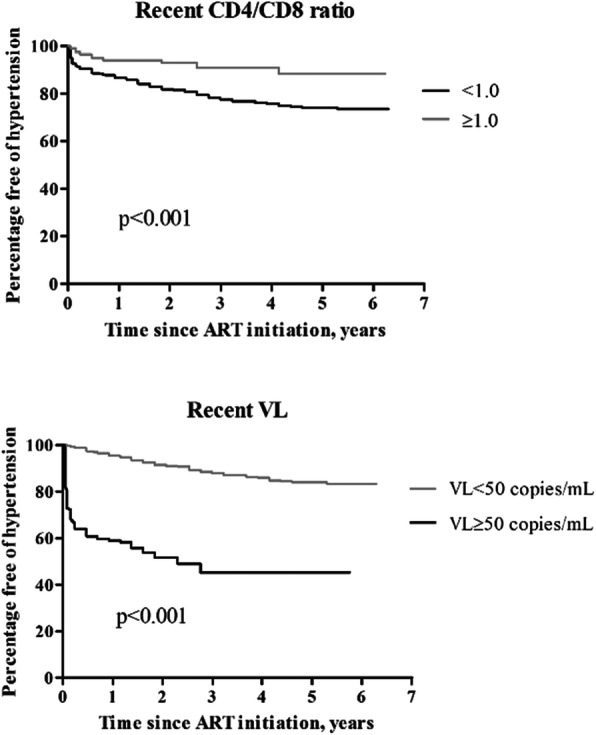
Kaplan-Meier survival estimates of incident hypertension. Abbreviations: ART, antiretroviral therapy; VL, HIV-1 viral load

Table 3 shows the results of our univariate and multivariable regression analyses. In the multivariable Cox regression model, for every 1 kg/m^2^ increase in BMI, we observed a 7% increase in the incidence of hypertension [adjusted HR (aHR) 1.07, 95% CI:1.01–1.13, *p* = 0.02]. Zidovudine exposure (aHR 0.15, 95% CI: 0.10–0.24, *p* < 0.001) and TDF exposure (aHR 0.13, 95% CI: 0.08–0.21, p < 0.001) correlated with a lower risk of hypertension. At the time of hypertension diagnosis or the last encounter (for those who did not develop hypertension), a lower recent CD4+/CD8+ ratio (aHR 0.14, 95% CI: 0.06–0.31, p < 0.001) and a higher recent VL (aHR 1.28, 95% CI: 1.08–1.51, *p* < 0.01) were also associated with incident hypertension (Fig. 2).

**Table 3:** Cox Regression Analysis of Association between Clinical Risk Factors and Incidence of Hypertension

*Covariate*	*Univariate model* *HR (95% CI)*	*P value*	*Multivariable model* *HR (95% CI)*	*P value*
Male	1.14 (0.80, 1.62)	0.47		
Age (per *year increase)*	1.04 (1.03, 1.05)	< 0.001	1.02 (1.00, 1.03)	0.06
BMI (per *kg/m*^*2*^*increase)*	1.09 (1.04, 1.14)	< 0.001	1.07 (1.01, 1.13)	0.02
Han ethnicity	1.27 (0.80, 2.02)	0.32		
Current smoking	0.84 (0.59, 1.21)	0.35		
Current alcohol use	0.83 (0.58, 1.18)	0.30		
eGFR (per *ml/min increase*)	0.99 (0.98, 1.00)	< 0.01	NA	NA
Diabetes	1.19 (0.61, 2.32)	0.62		
TC > 5.2 (*mmol/l*)	1.24 (0.79, 1.96)	0.36		
TG > 1.7 (*mmol/l*)	1.18 (0.86, 1.63)	0.31		
HDL-c < 1.0 (*mmol/l*)	1.22 (0.90, 1.67)	0.20		
LDL-c > 4.1(*mmol/l*)	1.42 (0.45, 4.45)	0.55		
Dyslipidemia	1.28 (0.95, 1.73)	0.11		
HBs Ag+	1.16 (0.74, 1.82)	0.51		
HCV-Ab+	1.69 (1.05, 2.72)	0.03	NA	NA
Route of HIV transmission			NA	NA
Heterosexual	0.99 (0.55, 1.80)	0.98		
MSM	1.21 (0.67, 2.17)	0.53		
Blood transfusion	4.12 (2.08, 8.19)	< 0.001		
Others	Reference			
Years since HIV diagnosis (per *year increase)*	1.06 (0.97, 1.14)	0.19		
Baseline CD4+ cell count (per *cells/mm*^*3*^*increase)*	0.999 (0.997, 1.000)	0.03	1.003 (1.001, 1.004)	< 0.01
Baseline HIV Viral load (per *log10 copies/ml increase)*	0.97 (0.74, 1.27)	0.82		
Stavudine exposure	1.92 (1.42, 2.60)	< 0.001	NA	NA
Zidovudine exposure	0.50 (0.36, 0.71)	< 0.001	0.15 (0.10, 0.24)	< 0.001
TDF exposure	0.46 (0.33, 0.64)	< 0.001	0.13 (0.08, 0.21)	< 0.001
Recent CD4+ cell count (per *cells/mm*^*3*^*increase)*	0.996 (0.995, 0.997)	< 0.001		
Recent CD4+/CD8+ ratio	0.09 (0.05, 0.17)	< 0.001	0.14 (0.06, 0.31)	< 0.001
Recent VL (per *log10 copies/ml increase)*	1.87 (1.65, 2.12)	< 0.001	1.28 (1.08, 1.51)	< 0.01

Abbreviations: *HR* Hazard ratio; *CI* confidence interval; *NA* not available; *BMI* body mass index; *TC* total cholesterol; *TG* triglycerides; *HDL-c* high-density lipoprotein cholesterol; *LDL-c* low-density lipoprotein cholesterol; *HBsAg+* hepatitis B surface antigen; *HCV Ab* hepatitis C antibody; *HIV* human immunodeficiency virus; *MSM* men who have sex with men; *TDF* tenofovir disoproxil fumarate; *VL* HIV-1 viral load

## Discussion

This study is the first to report incidence of hypertension among Chinese PLWH and to evaluate risk factors associated with incident hypertension in this population. We found that hypertension incidence was 7.6 (95% CI:6.5–8.7) per 100 PYs, and higher incidence was significantly associated with specific traditional (high BMI), and HIV-related risk factors (higher recent VL, lower recent CD4+/CD8+ ratio, lack of exposure of TDF or zidovudine).

While hypertension is commonly seen among PLWH, data conflict regarding whether hypertension is more prevalent among ART-naive PLWH compared with HIV-negative controls, as there is significant heterogeneity across different study designs [22]. The prevalence of hypertension observed among ART-naive PLWH in the present study was lower than that reported in the Chinese general population (26.9%), among a nationally representative sample of over 90,000 Chinese adults from 2007 to 2008 [23]. This might be attributable to younger age, lower BMI and prevalence of smoking among Chinese ART-naïve PLWH in the present study compared with the general population cohort, or to differences in other risk factors between the time periods during which the two cohorts were enrolled [12, 24]. The prevalence of hypertension observed in our study was also lower than that reported by Ding et al. among Chinese PLWH (23.8%), however that study was carried out in a single study site in Zhejiang province, and included both ART-naïve and ART-experienced PLWH [13].

By contrast, the incidence of hypertension in our cohort was slightly higher than that reported in the general Chinese population (7.6 vs. 5.2–5.3 per 100 PYs) [24, 25]. In terms of comparisons with incidence data from PLWH in other countries, an analysis of data from the Data collection on Adverse events of Anti-HIV Drugs (D:A:D) multi-cohort study from 1999 to 2003 found that the incidence of hypertension among PLWH in Europe, North America and Australia was 7.2 per 100 PYs [7]. However, in recent large studies from similar regions, the incidence of hypertension among PLWH was lower, and varied from 2.6 to 6.4 per 100 PYs [5, 9]. Data from Africa demonstrated incidence of hypertension was 11.2–12.0 per 100 PYs, which was higher than our present study [10, 11], which may reflect overall higher incidence of hypertension in persons of African descent. A population-based cohort study of 6814 middle-aged and older adults from the United States demonstrated that hypertension incidence was higher among African Americans (8.5 per 100 PYs) compared with Caucasians (5.7 per 100 PYs) and Chinese Americans (5.2 per 100 PYs) [25]. Therefore, the incidence of hypertension among patients in our study was slightly higher than general Chinese population and PLWH from Europe, North America and Australia, however lower than PLWH among Africa.

Interestingly, in our study, recent CD4+/CD8+ ratio among PLWH was associated with incident hypertension [Adjusted HR:0.14 (0.06, 0.31)]. CD4+/CD8+ ratio has been used in prior studies as a strong marker of immune activation and immune senescence [26], and a lower CD4+/CD8+ ratio during ART has been shown to predict residual plasma viremia of ≥1.0 copy/ml [27] among virologically suppressed PLWH. Further evidence has demonstrated that low CD4+/CD8+ ratios among PLWH are associated with neurocognitive disorders, lung cancer, pulmonary emphysema, and AIDS-related mortality [28–31]. No prior studies have examined the relationship between hypertension and CD4+/CD8+ ratios among PLWH. An abstract from the 2014 HIV Drug Therapy Glasgow Congress found that low CD4+/CD8+ ratio (< 0.8) predicted higher cardiovascular risk [32], and inversion of the CD4+/CD8+ ratio was associated with higher carotid intima-media thickness and arterial stiffness [33]. Among PLWH, chronic T-cell activation and release of cytokines promote renal sodium and water retention, vasoconstriction, and vascular remodeling, resulting in elevated blood pressure [34]. A few studies have also demonstrated a relationship between hypertension and inflammatory biomarkers (e.g. C-reactive protein, D-dimer and interleukin-6), immune activation and microbial translocation [35–37]. These data, combined with our findings of an association between low recent CD4+/CD8+ ratio and increased incidence of hypertension among PLWH, supports the need for additional studies aimed at confirming and better understanding the potential mechanisms behind this relationship.

A novel finding in this present study was that persistent HIV viremia was associated with incident hypertension among PLWH. Okeke et al. previously reported that HIV-related immunosuppression and ongoing viral replication may contribute to a higher risk of hypertension [8]; however in their study, no statistically significant differences were observed for the parameters of HIV viremia. Two studies from the United States and Europe also did not identify an association between HIV VL and the incidence of hypertension [4, 5]. However, these studies differed from ours with respect to the ethnic background of the participants and statistical approach. Prior studies have demonstrated the direct effects of HIV proteins, chronic inflammation induced by HIV-1 infection, and metabolic effects of ART contribute to endothelial dysfunction and atherosclerotic disease among PLWH [38, 39]. Endothelial dysfunction impairs endothelium-dependent vasodilatation, and causes alterations in the interactions between endothelium and leukocytes, thrombocytes and regulatory molecules, ultimately leading to hypertension [40].

Data regarding the relationship between specific antiretrovirals and hypertension have yielded conflicting results. Some studies have found that exposure to protease inhibitors, especially lopinavir/ritonavir, is associated with development of hypertension [41, 42], which may be attributable to activation of the adipocyte renin-angiotensin system [43]. Nduka et al. reported that stavudine-induced body composition change was associated with higher prevalence of hypertension [15]. Another longitudinal study demonstrated that prolonged exposure to both non-nucleoside reverse transcriptase inhibitors (NNRTI) and protease inhibitors was a risk factor of hypertension [44]. Several potential mechanisms have been hypothesized to explain the association between ART and hypertension, including gain in body weight, immune reconstitution, and endothelial dysfunction [41]. One animal model study showed zidovudine could increase blood pressure and promote cardiovascular damage through a NAD(P)H oxidase-dependent mechanism [45]. By contrast, other studies have found no association between exposure to protease inhibitors, stavudine and NNRTIs on the development of hypertension [5, 8, 11], and data from the D:A:D study demonstrated cumulative exposure to NNRTIs was associated with a decreased risk of incident hypertension [7]. In the present study, stavudine was not found to be associated with incident hypertension, possibly due to the relatively short length of exposure of stavudine and fewer body composition changes [16]. Our data also demonstrated TDF or zidovudine exposure were protective factors for incident hypertension among PLWH, which is discordant with the aforementioned studies [11, 42]. However, a retrospective longitudinal study from Brazil found that cumulative exposure of TDF or zidovudine were associated with lower cardiovascular event risk [46], which was consistent with our finding. The nuances of the relationship between ART and hypertension require further research.

In our univariate analysis, baseline CD4+ cell count was not associated with hypertension incidence [Crude HR:0.999 (0.997,1.000)], however in the adjusted model, baseline CD4+ cell count was associated with hypertension incidence [Adjusted HR:1.004 (1.002,1.006)]. Given the weak magnitude of the relationships observed in this regard, we feel this finding does not likely carry strong clinical implications for our study population and should not be over-interpreted.

Our study has a few limitations. First, our study population represents patients enrolled in two multi-center prospective studies of treatment-naïve patients initiating government-sponsored, currently available ART regimens at the time of enrollment. Therefore, our findings may be influenced by differences in the two studies. However, we did not detect a significant difference in the incidence rates between patients in the two groups, and given the patients were recruited from study sites within the same research network there was a high degree of consistency in the enrollment criteria and technique for study evaluations. We also acknowledge that the sample does not fully represent the entire population of patients with HIV in China, as participants were able to meet criteria for entry into the study and maintain follow-up care. However, if anything, we expect that this would underestimate the magnitude of our findings. Furthermore, while our sample size was smaller compared with studies from European and the United States [5, 7–9, 11], it represents the largest study to date in Asia to explore this important issue. In addition, it is possible that a few people classified as having hypertension based on taking an antihypertension medication maybe have been misclassified, as these medications may be used to treat other conditions. Finally, the follow-up period in the present study is comparatively short, and therefore continued follow up is needed to understand how incidence rates change after longer periods of exposure to ART.

## Conclusions

Hypertension is a major modifiable risk factor for cardiovascular disease, an important cause of mortality among PLWH. We found that incidence of hypertension was high compared with rates among the general population. Recent low CD4+/CD8+ ratio and detectable HIV viremia were associated with incident hypertension, while receipt of ART was associated with reduced risk. Hypertension may be mitigated, in part, by excellent HIV care, including viral suppression with ART.

## Data Availability

The datasets used and/or analyzed during the current study are available from the corresponding author on reasonable request.

## Abbreviations

aHR: Adjusted hazard ratio
ART: Antiretroviral therapy
AZT: Zidovudine
BMI: Body mass index
CACT: China AIDS Clinical Trial
CI: Confidence interval
d4T: Stavudine
EFV: Efavirenz
eGFR: Estimated glomerular filtration rate
HBsAg: Hepatitis B virus surface antigen
HBV: Hepatitis B virus
HCV: Hepatitis C virus
HCV Ab: Hepatitis C virus antibody
HDL-c: High-density lipoprotein cholesterol
HR: Hazard ratios
MSM: Men who have sex with men
NNRTI: Non-nucleoside reverse transcriptase inhibitors
NVP: Nevirapine
PLWH: Persons living with HIV
PUMCH: Peking Union Medical College Hospital
PYs: Person-years
3TC: Lamivudine
TDF: Tenofovir disoproxil fumarate
VL: Viral load
